# Glycated hemoglobin follow-up of sis-hiperdia (Muriaé-Minas Gerais, Brazil)

**DOI:** 10.1186/1758-5996-7-S1-A47

**Published:** 2015-11-11

**Authors:** Cláudio César Gusman Matias de Oliveira, Cláudio Rogério Matias de Oliveira, Gustavo Gusman Matias de Oliveira, Bruno Almeida Rocha Maciel, Matheus Sarabion Vilela Pereira, Vilma Aparecida Ferraz

**Affiliations:** 1 Universidade Presidente Antonio Carlos, Muriaé, Brazil

## Background

The glycated hemoglobin (HbA1C) test, essential for diabetic patients follow-up, measures the concentrations of glycated hemoglobin in the blood, which can indicate, as they increase in proportion to glucose rates, the possibility of complications of the disease. Furthermore, the test points to the risk of Type 2 diabetes mellitus (T2D) development, for concentration values between 5.7% and 6.4%, and confirms its diagnosis if values exceed 6.5% in two different tests. In T2D patients, values above 7% imply heightened risk of microvascular complications and neuropathy. Several factors in the patients' lifestyle affect these values, such as the practice of physical activities, diet and weight control.

## Objective

To evaluate the progress of T2D patients treated in the institution between January of 2013 and January of 2015, noting the HbA1C control levels.

## Material and methods

Review of 361 medical records of the institution, collection and statistical analysis of data. Patients with values of HbA1C below 9%, who are not included in the institution's system, and those who did not return for follow-up appointments, have been excluded from the study. The patients' age, gender and ethnicity were not taken into account.

## Results

Of the 361 evaluated patients, 244 (67.59%) showed a decrease in the values of HbA1C; 03 (0.83%) maintained at the same values; 23 (6.37%) exhibited an increase in the values; and only 35 (9.69%) reached values considered as adequate (<7%) at the end of the follow-up. Many patients, 56 (15.51%), abandoned the follow-up after the first appointment.

## Conclusion

The follow-up and treatment provided to T2D patients managed to reduce their HbA1C levels in most cases, although small the fraction of those who attained Results considered ideal. The number of patients who exhibited the opposite result, along with those who discontinued the treatment, poses the question of whether through better scrutiny of patients' conditions their chances of success might rise.

